# Gut microbiota influence immunotherapy responses: mechanisms and therapeutic strategies

**DOI:** 10.1186/s13045-022-01273-9

**Published:** 2022-04-29

**Authors:** Yuting Lu, Xiangliang Yuan, Miao Wang, Zhihao He, Hongzhong Li, Ji Wang, Qin Li

**Affiliations:** 1 Department of Oncology, Beijing Friendship Hospital, Capital Medical University, Beijing, 100050 China; 2 Department of Laboratory Medicine, Ruijin Hospital, Shanghai Jiao Tong University School of Medicine, Shanghai, 200025 China; 3 Chongqing Key Laboratory of Molecular Oncology and Epigenetics, The First Affiliated Hospital of Chongqing Medical University, Chongqing, 400010 China; 4 National Institute of TCM Constitution and Preventive Medicine, Beijing University of Chinese Medicine, Beijing, 100029 China

**Keywords:** Cancer immunotherapy, Immune checkpoint inhibitors, Gut microbiota, Microbiota-derived metabolites, Therapeutic strategies

## Abstract

The gut microbiota have long been recognized to play a key role in human health and disease. Currently, several lines of evidence from preclinical to clinical research have gradually established that the gut microbiota can modulate antitumor immunity and affect the efficacy of cancer immunotherapies, especially immune checkpoint inhibitors (ICIs). Deciphering the underlying mechanisms reveals that the gut microbiota reprogram the immunity of the tumor microenvironment (TME) by engaging innate and/or adaptive immune cells. Notably, one of the primary modes by which the gut microbiota modulate antitumor immunity is by means of metabolites, which are small molecules that could spread from their initial location of the gut and impact local and systemic antitumor immune response to promote ICI efficiency. Mechanistic exploration provides novel insights for developing rational microbiota-based therapeutic strategies by manipulating gut microbiota, such as fecal microbiota transplantation (FMT), probiotics, engineered microbiomes, and specific microbial metabolites, to augment the efficacy of ICI and advance the age utilization of microbiota precision medicine.

## Background

The microbiota in the gastrointestinal tract, which produces a myriad of small molecules and metabolites, play an essential role in multiple human physiological processes, including metabolism, inflammation, immunity, and neurology [1–5]. The function of the microbiota and its metabolites in modulating local and systemic immune responses has led to the emergence of research on the effects of the cancer-immune system and immune checkpoint inhibitor (ICI) therapeutic response [6–10]. The widespread variability in the gut microbiota across adult individuals is another reason to consider the gut microbiome as a potential source of phenotypic variability in cancer progression and ICI treatment outcomes [11].

Mounting evidence supports the role of the gut microbiota in the ICI response in preclinical and clinical studies [12–17]. ICI immunotherapy has improved traditional cancer therapeutic medicine and has enabled breakthroughs in the treatment of solid metastatic malignancies [18–21]. ICIs unleash immune brake responses and effectively inhibit tumor immune escape by targeting programmed cell death 1 (PD-1) and its ligand (PD-L1), lymphocyte-activating gene-3 (LAG3), cytotoxic T lymphocyte-associated antigen-4 (CTLA-4), and other targets [18, 22, 23]. However, responses to ICI therapy are heterogeneous and not robust, with patient objective response rates (ORRs) of only 10–30% [24–27]. Manipulation of the gut microbiota provides novel insight for improving the antitumor immune response and expanding ICI efficacy.

Nonetheless, the potential molecular mechanisms of the influence of the gut microbiome and its metabolites on ICI efficacy are still poorly understood in some cancer types. In this review, we have summarized studies of the influence of the gut microbiome on ICI efficacy and discussed the mechanisms of microbiota cross talk with innate and adaptive immune cells to ameliorate ICI responses. We will specifically highlight the mechanisms of microbiota-derived metabolites and molecule-mediated antitumor immune responses to ICI. Finally, we will review the therapeutic strategies and ongoing trials investigating manipulation of gut microbiota to improve ICI efficacy.

## Gut microbiota and efficacy of immunotherapy: from discovery to applications

Early studies have shown that gut microbiota could stimulate antitumor immune responses by modulating CD8^+^ T cells [28], T helper 1 (Th1) [29], and tumor-associated myeloid cells [30]. Indeed, the effects of cancer therapy were attenuated in antibiotic-treated or germ-free mouse models and were affected by special gut microbiota species. Importantly, landmark publications in 2015 in mouse models first linked the gut microbiota to ICI responses [16, 17]. Gut microbiota composition influenced anti-PD-L1 therapy responses, and the difference in responses was eliminated upon FMT or cohousing [16]. Oral administration of *Bifidobacterium* restored the antitumor efficacy of PD-L1 blockade by enhancing dendritic cell (DC) maturation and increasing CD8^+^ T cell priming and accumulation in the tumor microenvironment (TME) [16]. Another contemporary parallel study on anti-CTLA-4 therapy suggested that antibiotics dampen the antitumor effect of ICI, and supplementation with *Bacteroides fragilis* in germ-free or antibiotic-treated melanoma mice could augment anti-CTLA-4 therapeutic efficacy [17]. The microbiota-dependent antitumor effect is dependent on eliciting Th1-cell activation in the tumor draining lymph node and inducing maturation of intratumoral DCs [17].

Human studies published in Science side by side in 2018 complemented these mouse studies. The three studies all demonstrated that gut microbiota composition and diversity were predictive of response to ICI immunotherapy [31–33]. A similar finding was that fecal microbiota transplantation (FMT) from ICI responding patients to germ-free or antibiotic-treated mice could improve tumor control and ameliorate responses to ICI, whereas FMT from non-responders failed to do so [31–33]. In non-small cell lung cancer (NSCLC) and renal cell carcinoma (RCC), patients with a higher diversity of bacteria were more sensitive to anti-PD-1 therapy [31]. Oral supplementation with *Akkermansia muciniphila* (*A. muciniphila*) after FMT from ICI non-responders restored anti-PD-1 therapy responses [31]. In melanoma patients, the diversity and composition of the gut microbiota were positively correlated with anti-PD-1 therapy responses [32]. Mostly, ICI responding patients with a higher abundance of *Faecalibacterium* and *Ruminococcaceae* in the gut displayed increased numbers of CD4^+^ T cells and CD8^+^ T cells in the periphery [32]. Another study involving patients with metastatic melanoma indicated that *Bifidobacterium longum*, *Collinsella aerofaciens*, and *Enterococcus faecium* were more abundant in the baseline feces of responders [33].

**Fig. 1:**
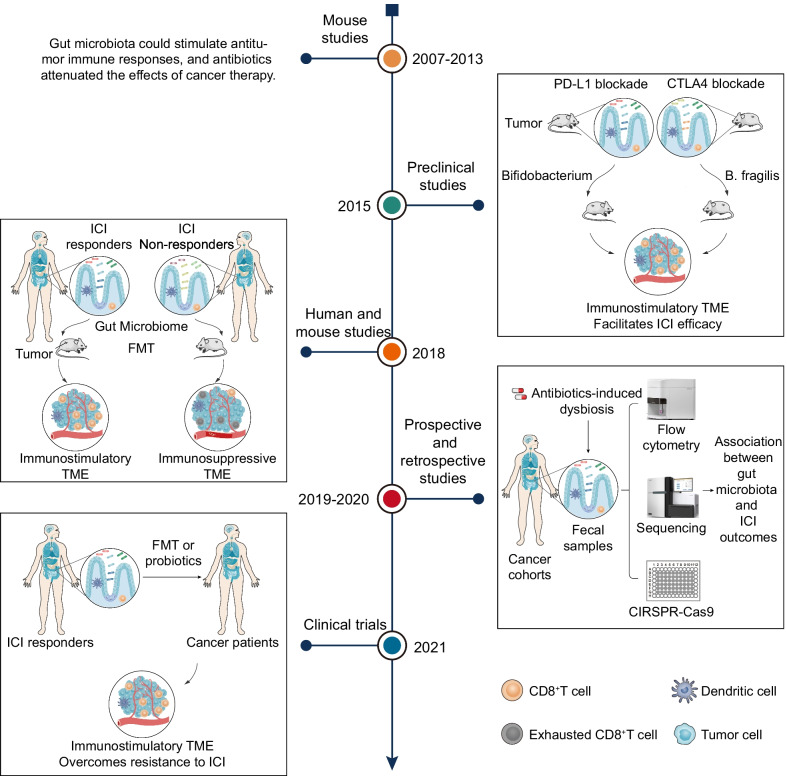
Timeline of gut microbiota and ICI efficacy: from discovery to application. From 2007 to 2013, mouse studies showed that the gut microbiota could stimulate antitumor immune responses**.** In 2015, two preclinical mouse studies first linked the gut microbiota to ICI responses. In 2018, mouse and human studies demonstrated that gut microbiota composition and diversity were predictive of the response to ICI immunotherapy. FMT from ICI responding patients to germ-free or antibiotic-treated mice could improve tumor control and ameliorate responses to ICI. From 2019 to 2020, prospective studies confirmed a significant association between gut microbiota and ICI outcomes in advanced solid tumors. Retrospective studies have implicated that antibiotics are associated with decreased survival and attenuated response to ICI. In 2021, two clinical trials found that FMT from ICI responders combined with anti-PD-1 therapy overcame resistance to PD-1 blockade in melanoma patients

Prospective studies confirmed a significant association between gut microbiota and ICI outcomes in NSCLC, hepatocellular carcinoma (HCC), melanoma, and RCC patients from 2019 to 2020 [34–38]. At the same time, retrospective studies have implicated that antibiotics were associated with decreased survival and attenuated response to ICI in patients with advanced solid tumors [39–44], supporting a causal link between antibiotic-induced dysbiosis and poor therapeutic efficacy of ICI. Furthermore, two clinical trials unexpectedly found that FMT from ICI responders combined with anti-PD-1 therapy overcame resistance to PD-1 blockade in melanoma patients in 2021 [14, 15]. The timeline of gut microbiota and ICI efficacy is summarized in Fig. 1.

In addition to modulating ICI immunotherapy, gut microbiota can influence adoptive T cell transfer (ACT) immunotherapy, CpG-oligodeoxynucleotide (CpG-ODN) immunotherapy, and cell-based immunotherapy. Antibiotic exposure decreased the efficacy of ACT therapy in mice [28, 45], while bacterial LPS supplementation restored the therapeutic effect via toll-like receptor (TLR) 4 signaling [28]. Another study demonstrated that the gut microbiota maintained ACT therapeutic efficacy by increasing the abundance of CD8α^+^ DCs, and upregulation of interleukin (IL)-1 [46]. In CpG-ODN immunotherapy, the gut microbiota activate TLR4, which directly or indirectly initiates the TLR9-dependent response of tumor-associated myeloid cells to CpG-ODNs [30]. The efficacy of CpG-ODN is abolished in GF and antibiotic-exposed mice by impairing the production of tumor necrosis factor (TNF) and IL-12 [30]. The gut microbiota also affect cell-based immunotherapy. Gut microbiota-mediated metabolism of bile acid increased the abundance of CXCR6^+^ natural killer T (NKT) cells in the liver and played an antitumor role in hepatocellular carcinomas [47].

## The gut microbiota remodel the TME to improve ICI efficacy

**Fig. 2:**
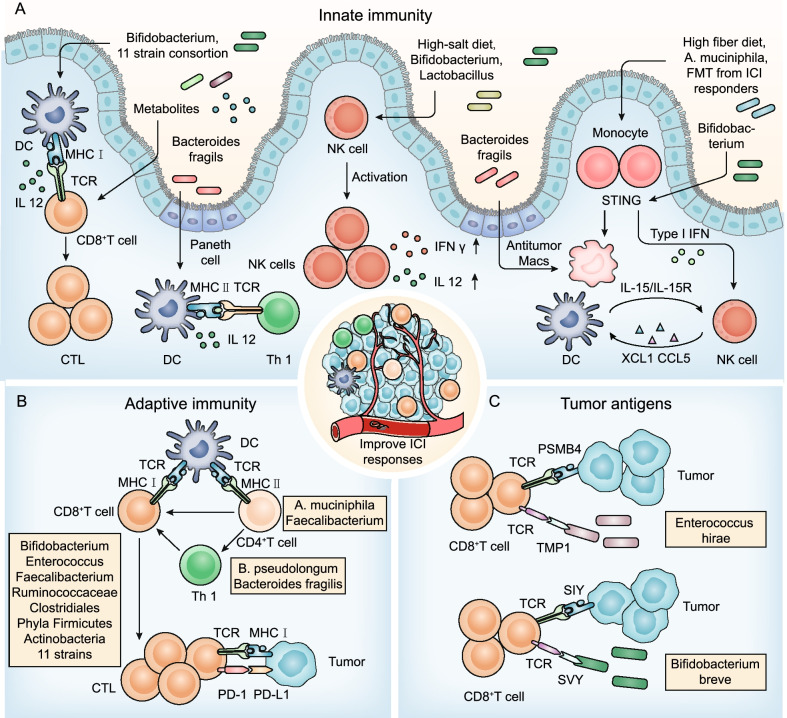
The gut microbiota modulate innate immunity, adaptive immunity, and tumor antigens to improve ICI responses. **A** Innate immunity. DCs: *Bifidobacterium*, eleven strains and their metabolites, and *Bacteroides fragilis* promote DC maturation or activation and subsequent activation of CD8^+^ T cells and Th1 cells. NK cells: *Lactobacillus plantarum* increases NK cell activation; a high-salt diet increases intestinal permeability and localization of intratumoral *Bifidobacterium* and enhances NK cell activation to induce antitumor immunity. Monocyte: Feeding a high-fiber diet, monocolonization with cdAMP-producing *A. muciniphila* or transferring fecal microbiota from ICI responders can trigger the monocyte-IFN-I-NK-cell-DC cascade; *Bifidobacterium* facilitates CD47-based immunotherapy in a STING signaling and IFN-I-dependent fashion; *Bacteroides fragilis* induces macrophage phenotype polarization to M1. **B** Adaptive immunity. CD8^+^ T cells: *Bifidobacterium*, *Enterococcus*, *Faecalibacterium*, *Ruminococcus*, and *Clostridiales* promote CD8^+^ T cell infiltrates in tumor tissues; *Phyla Firmicutes* and *Actinobacteria* improve the activation of CD56^+^CD8^+^ T cells in the peripheral blood of ICI responders; and eleven strains increase the proportion of effector IFNγ^+^CD8^+^ T cells in the systemic circulation. CD4^+^ T cells: *B. pseudolongum* and *Bacteroides fragilis* stimulate Th1 immune responses; *A. muciniphila* triggers CCR9^+^CXCR3^+^CD4^+^ T lymphocyte recruitment into tumor beds; and *Faecalibacterium* increases the CD4^+^ T cell proportion. **C** Tumor cross-antigen. The gut microbiota increase the immunogenicity of tumor cells by providing tumor cross-antigens to ameliorate the efficacy of ICIs, including the antigen epitope TMP1 and the antigen epitope SVY

Studies have shown that the gut microbiota modulate the ICI response, and detailed mechanistic exploring into the specific bacterial species and microbial metabolites on ICI is necessary. The gut microbiota can modulate innate and adaptive immunity and influence antitumor immune responses in the TME [2, 48]. Complex mechanisms by which specific bacterial species reprogram the TME to improve ICI efficacy in the context of immunity will be discussed herein (Table 1 and Fig. 2).

**Table 1:** Studies on gut microbiota target innate and adaptive immune cells to promote ICI efficacy

Year	Cancer types	ICI	Beneficial gut microbiota	Interventions factors and/or biological effects	References
2015	Melanoma	PD-L1 inhibitor	*Bifidobacterium*	DCs and CD8 + T cells	[16]
2015	Melanoma	CTLA4 inhibitor	*Bacteroides fragilis*	Tumor draining lymph nodes: Th1; TME: DCs	[17]
2018	NSCLC, RCC, Urothelial carcinoma	PD-1 inhibitor, CTLA4 inhibitor	*Akkermansia muciniphila*	TME: IL-12 and CCR9 + CXCR3 + CD4 + T lymphocytes	[31]
2018	Melanoma	PD-1 inhibitor	*Faecalibacterium*, *Ruminococcaceae*, Clostridiales	Mice receiving R-FMT: Increased innate effector cells and decreased suppressive myeloid cells; Mice receiving NR-FMT: Increased RORγT^+^ T helper 17 cells	[32]
2018	Melanoma	PD-L1 inhibitor	*Bifidobacterium longum*, *Collinsella aerofaciens*, *Enterococcus faecium*	Mice receiving R-FMT: Augmented T cell responses	[33]
2017	Melanoma	CTLA4 inhibitor	*Faecalibacterium*	CD4 + T cells and CD25	[49]
2019	Adenocarcinoma, melanoma	PD-1 inhibitor	Eleven strains	IFNγ + CD8 T cell, CD103 + DC, and MHC Ia	[50]
2020	CRC, Intestinal cancer, Bladder cancer, melanoma	PD-L1 inhibitor, CTLA4 inhibitor	*Bifidobacterium pseudolongum, Akkermansia muciniphila*, *Lactobacillus johnsonii*, *Olsenella species*	DCs and Th1; Inosine: A_2A_R on Th1	[51]
2020	Colon cancer, T cell lymphoma	CD47 inhibitor	*Bifidobacterium*	STING signaling and DCs	[52]
2021	Melanoma	PD-1 inhibitor	*Enterococcaceae*, *Enterococcus*, *Streptococcus australis*	FMT-R patients: TME: CD8 + T cell; Gut: APC	[15]
2021	Melanoma	PD-1 inhibitor	*Lachnospiraceae*, *Ruminococcaceae families*, *Bifidobacteriaceae*, *Coriobacteriaceae families*	FMT-R patients: PBMCs: CD8 + T cells and MAIT cells; TME: CD8 + T cells, HLA II, CD74 and GZMK FMT-NR patients: Increased myeloid cells and CD4 + regulatory T cells	[14]
2021	Lymphoma, Colon carcinoma, Melanoma, Breast carcinoma	ICI	A high-fiber diet, *Akkermansia muciniphila*	Monocytes, Macrophages, NK cells, DCs, Type I IFN, and STING	[53]

APC: antigen presenting cell; A_2A_R: adenosine 2A receptor; CRC: colorectal cancer; CTLA4: cytotoxic T lymphocyte-associated antigen 4; DC: dendritic cell; FMT: fecal microbiota transplant; FMT-R: responders to fecal microbiota transplant treatment; FMT-NR: non-responders to fecal microbiota transplant treatment; GZMK: granzyme K; ICI: immune checkpoint inhibitor; IFN: interferon; MAIT: mucosal-associated invariant T; MHC: major histocompatibility; NK: natural killer; NSCLC: non-small cell lung cancer; NR-FMT: fecal microbiota transplants from non-responders to immune checkpoint inhibitor; PBMCs: peripheral blood mononuclear cells; PD-1: programmed cell death 1; PD-L1: programmed cell death ligand 1; RCC: renal cell carcinoma; R-FMT: fecal microbiota transplants from responders to immune checkpoint inhibitor; STING: stimulator of interferon gene; Th1: T helper 1; and TME: tumor microenvironment

### Gut microbiota modulate innate immunity to ameliorate ICI responses

#### DCs

DCs are a group of special antigen-presenting cells that play an essential role in T cell activation and antitumor immunity [54–57]. Gut microbiota antigens or metabolites with immunomodulators were used to mobilize and activate DCs to reverse immature DC-induced immune tolerance [58] (Fig. 2A). For instance, oral administration of *Bifidobacterium* increased DC activation, which in turn supported improved tumor-specific CD8^+^ T cell responses and restored the therapeutic efficacy of anti-PD-L1 therapy in mice with an “unfavorable” gut microbiota [16]. Eleven strains combined with ICI robustly induced interferon (IFN) γ^+^ CD8^+^ T cells to inhibit tumor growth in a manner dependent on lamina propria cDC1 and major histocompatibility complex (MHC) class I [50]. *Bacteroides fragilis* enhanced the antitumor effect of CTLA-4 blockade by triggering DC maturation and stimulating IL-12-dependent Th1 cell immune responses [17]. Furthermore, vancomycin-mediated modulation of gut microbiome composition improved the activities of antitumor-specific effector T cells by increasing cDC1 and upregulating IL-12 [46].

### Monocytes/macrophages

Skews in IFN-I and mononuclear phagocyte levels contribute to immune dysregulation and an immunosuppressive TME [59]. Microbiota-induced IFN-I signaling mediates the transition from innate immunity to adaptive immunity [7, 53, 60] (Fig. 2A). Microbiota-derived stimulator of interferon genes (STING) agonists (such as c-di-AMP) induce IFN-I signaling by intratumoral monocytes, which shift mononuclear phagocytes toward antitumor macrophages (Macs) and trigger monocyte-IFN-I-natural killer (NK) cell-DC cross talk [53]. Notably, feeding a high-fiber diet, monocolonization with cdAMP-producing *A. muciniphila* or transferring fecal microbiota from ICI responders improved the antitumor responses and ICI efficacy [53]. Similarly, it has been demonstrated that *Bifidobacterium* preferentially colonizes the tumor site and facilitates CD47-based immunotherapy in a STING signaling-and IFN-I-dependent fashion [52]. In addition, *Bacteroides fragilis* induced macrophage phenotype polarization to M1 and upregulated CD80 and CD86 expression on the cell, promoting innate immunity [61].

### NK cells

It has been demonstrated that NK cells can regulate DC and CD8^+^ T cell abundance in the TME and influence responses to ICIs [54, 62–64]. Recently, an increasing number of studies have found an interplay between NK cells and gut microbiota (Fig. 2A). NSCLC patients with high microbial diversity had a higher abundance of unique memory CD8^+^ T cells and NK cell subsets in the periphery in response to PD-1 blockade [34]. *Lactobacillus plantarum* effectively increased expression of the natural cytotoxic receptor (NCR) protein and promoted NK cell activation to trigger innate immunity [65]. Interestingly, the suboptimal dose of PD-1 blockade combined with a high-salt diet significantly inhibited tumor growth in mice [66]. Mechanistic studies found that a high-salt diet increased intestinal permeability and the localization of intratumoral *Bifidobacterium*, which enhanced NK cell activation to induce antitumor immunity [66].

### Gut microbiota modulate adaptive immunity to improve ICI responses

#### CD8^+^ T cells

Multiple publications have confirmed that specific gut microbiota induce CD8^+^ T cells in the systemic circulation or the TME (Fig. 2B). For example, melanoma patients with a high relative abundance of favorable microbiota, including *Clostridiales*, *Ruminococcaceae*, or *Faecalibacterium*, increased antigen presentation and improved effector CD4^+^ T cell and CD8^+^ T cell function in the peripheral blood and TME to ameliorate the antitumor efficacy of ICI [32]. Evidence from a clinical trial has implicated that the *phyla Firmicutes* and *Actinobacteria* were enriched in FMT combined with PD-1 blockade responders [14]. Combined FMT and PD-1 blockade stimulated mucosal-associated invariant T (MAIT) cells and CD56^+^CD8^+^ T cells in peripheral blood mononuclear cells (PBMCs) and upregulated expression of the human leucocyte antigen (HLA) class II genes CD74 and GZMK on CD8^+^ T cells at tumor sites [14]. In parallel, after using FMT combined with PD-1 blockade promoted a high relative abundance of *Enterococcus* in refractory metastatic melanoma and led to increased intratumoral CD8^+^ T cell infiltration and tumor cell necrosis [15]. Furthermore, *Bifidobacterium* and eleven strains could also increase the abundance of CD8^+^ T cells reliant on DCs to improve ICI therapy efficacy [16, 50].

#### CD4^+^ T cells

In mouse models, *B. pseudolongum* promoted Th1 transcriptional differentiation and antitumor immune responses to improve ICI efficacy mainly through the gut microbial metabolite inosine [51]. *Bacteroides fragilis* stimulated IL-12-dependent Th1 immune responses by facilitating the mobilization of lamina propria DCs, which restored the immune responses to ICI [17]. In addition, oral supplementation with *A. muciniphila* in FMT non-responsive mice recovered anti-PD-1 responses by triggering CCR9^+^CXCR3^+^CD4^+^ T lymphocyte recruitment into tumor beds [31]. In human patients, *Faecalibacterium* increased the CD4^+^ T cell proportion and serum CD25 production and reduced the Treg cell proportion in peripheral blood, which induced the long-term clinical benefit of ipilimumab [49] (Fig. 2B).

### Gut microbiota modulate the immunogenicity of tumor cells to improve ICI responses

The decrease in tumor immunogenicity is an essential mechanism by which tumor cells resist T cell killing. On the one hand, the gut microbiota can directly enhance the innate immunogenicity of tumor cells by acting on UBA6 on the tumor cell surface to augment ICI responses [67]. On the other hand, the gut microbiota can indirectly increase the immunogenicity of tumor cells by providing tumor cross-antigens to promote the efficacy of ICI [50, 68, 69]. Some cross-reactivity between antigens expressed in gut microbes and tumor cells has been identified (Fig. 2C). The antigenic epitope tail length tape measure protein 1 (TMP1) in the genome of bacteriophage *Enterococcus hirae* had high similarity with the proteasome subunit beta type-4 (PSMB4) tumor antigen [70]. They activated CD8^+^ T cells simultaneously and improved the efficacy of PD-1 blockade therapy [70]. It has been demonstrated that the antigen epitope SVYRYYGL (SVY) expressed in the commensal bacterium *Bifidobacterium breve* was similar to the tumor-expressed antigen epitope SIYRYYGL (SIY), resulting in SVY-specific T cells recognizing SIY and inhibiting tumor growth [71].

## Gut microbial metabolite-mediated antitumor immune responses to ICI

Notably, one of the primary modes by which the gut microbiota modulate antitumor immunity is by means of metabolites. The gut microbiota synthesize or transform a myriad of metabolites, which are small molecules that can spread from their original location in the gut and impact the local and systemic antitumor immune response to promote ICI efficacy [14, 72]. Accordingly, we further explored the mechanisms of the different gut microbial metabolites and other features of gut microbial signatures mediating antitumor immune responses.

### Inosine

**Fig. 3:**
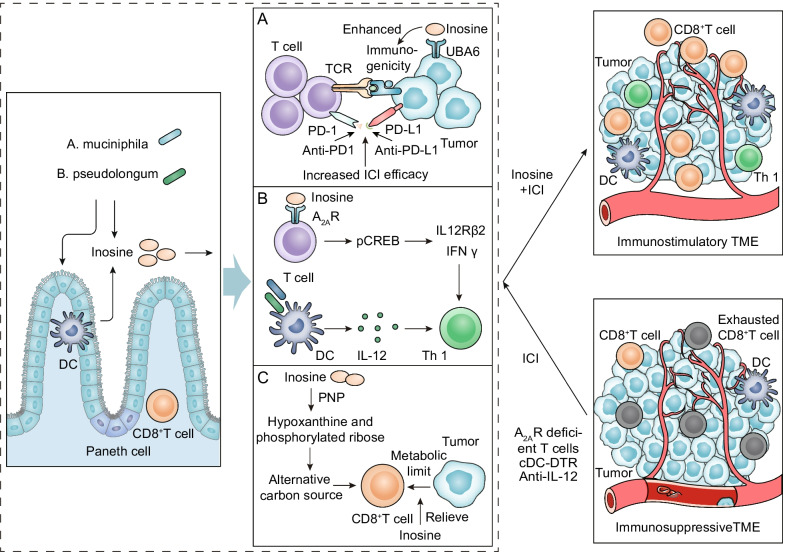
Potential mechanisms by which the gut microbial metabolite inosine facilitates the efficacy of ICI. Inosine, purine metabolite of gut microbiota *A. muciniphila* and *B. pseudolongum*, combined with in ICI therapy exert synergistic antitumor effects. **A** Inosine increases the immunogenicity of tumor cells. Inosine can improve the ability of tumor cells to present tumor antigens so that cytotoxic immune cells can easily recognize and kill tumor cells. **B** Inosine promotes immune cell activation. Inosine could enhance ICI efficacy by acting on A_2A_R on T lymphocytes. It stimulates the phosphorylation of cAMP response element-binding protein (pCREB) through the inosine-A_2A_R-cAMP-PKA signaling pathway, which can upregulate IL12Rβ2 and IFNγ transcription and promote Th1-cell differentiation and accumulation in the TME. **C** Inosine provides an alternative carbon source for CD8^+^ T cells. Inosine can be used as an alternative carbon source for CD8^+^ T cells when glucose is limited and relieves the restrictions on CD8^+^ T cells energy metabolism in tumor cells

Inosine, the purine metabolite of *Akkermansia muciniphila* and *Bifidobacterium pseudolongum* (*B. pseudolongum*), plays a vital role in improving the efficacy of ICI [51]. Inosine is a normal metabolite of the human body and participates in nucleic acid metabolism, energy metabolism, and protein synthesis in a physiological state. It can activate immune cells and stimulate metabolism. In the past, it has been shown that inosine has immunosuppressive effects [73, 74]. However, in recent years, more studies have found that inosine could reprogram the TME and improve the response to ICI therapy [51, 75, 76]. Current studies indicate that inosine affects the efficacy of ICI mainly through the following mechanisms (Fig. 3).

Inosine increases the immunogenicity of tumor cells. A research group found that inosine could significantly enhance the ability of tumor cells to present tumor antigens, so cytotoxic immune cells could easily recognize and kill tumor cells, achieving an antitumor effect [77]. Further mechanistic studies showed that activation of the IFNγ and TNFα signaling pathways was significantly increased in tumor cells treated with inosine. IFNγ can activate the cytotoxicity of tumor-specific T cells and NK cells through promoting their release of perforin and granzyme, and IFNγ can heighten antigen presentation to promote inosine-mediated antitumor effects [78]. Our recent work demonstrated that inosine sensitized tumor cells to T cell-mediated cytotoxicity by directly binding and inhibiting the ubiquitin-activating enzyme UBA6 to amplify tumor-intrinsic immunogenicity and enhance ICI efficacy [67] (Fig. 3A).

Inosine promotes immune cell activation. The gut microbial metabolite inosine could improve the efficacy of ICI by acting on adenosine 2A receptor (A_2A_R) on T lymphocytes in intestinal cancer, bladder cancer, and melanoma mouse models [51]. The inosine-A_2A_R-cAMP-PKA signaling cascade led to the phosphorylation of cAMP response element-binding protein (pCREB), which upregulated IL12Rβ2 and IFNγ transcription and promoted Th1-cell differentiation and accumulation in the TME [51]. Interestingly, the in vivo antitumor effects of ICI combined with inosine required a costimulus, such as CpG and IL-12 [51] (Fig. 3B). Inosine can also enhance immune responses mediated by phytohemagglutinin (PHA), increase tumor antigen levels, and strengthen T lymphocyte differentiation and proliferation [75, 79]. Furthermore, inosine stimulated B lymphocyte differentiation and antibody production by activating macrophages, exerting antiviral and antitumor actions [75, 79].

Inosine provides an alternative carbon source for CD8^+^ T cells. Oncogenic signaling pathway activation reprograms the metabolism of cancer cells [80]. The high metabolic demand of cancer cells can limit the capacity of effector T cells by reducing the levels of available nutrients and producing immunosuppressive metabolites [81, 82]. Inosine could be employed as an alternative carbon source for CD8^+^ T cells under energy metabolism limits to support the growth and capacity of CD8^+^ T cells [75] (Fig. 3C). T cells metabolize inosine into hypoxanthine and phosphorylated ribose by purine nucleoside phosphorylase (PNP) [75] (Fig. 3C). Importantly, the ribosomal subunit of inosine enters the central metabolic pathway, providing ATP and biosynthetic precursors for the glycolytic pathway and the pentose phosphate pathway (PPP) [75].

### Short-chain fatty acids (SCFAs)

**Fig. 4:**
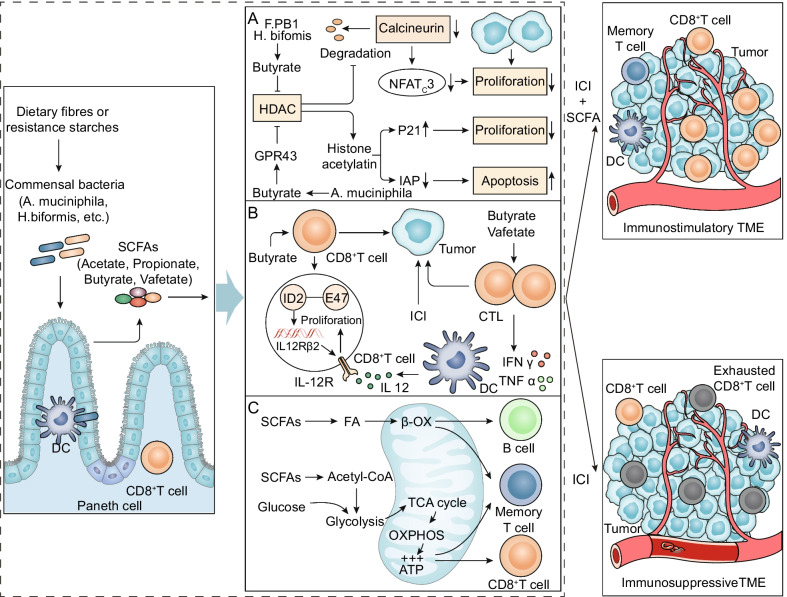
Potential mechanisms by which the gut microbial metabolite SCFAs augment the efficacy of ICI. **A** SCFAs inhibit the proliferation and induce the apoptosis of cancer cells. The butyric acid of SCFAs, a metabolite of *Faecalibaculum rodentium* PB1 and *H. biformis*, inhibits the activity of HDAC and the calcineurin-mediated activation of NFATc3 transcription factor, thereby blocking the proliferation of tumor cells. Propionic acid produced by *A. muciniphila* activates the cell cycle inhibitor p21 through GPR43 and downregulates the IAP inhibitor, which inhibits cancer cell proliferation, induces apoptosis, and improves the antitumor effect of ICI. **B** SCFAs improve the antitumor immune response. Butyrate can directly enhance CD8^+^ T cell antitumor cytotoxicity by inducing ID2 expression in CD8^+^ T cells through IL-12 signaling. Valeric acid and butyric acid of SCFAs promote expression of effector molecules such as IFNγ and TNFα and enhance the antitumor effects of CTLs. **C** SCFAs provide energy for immune cells. SCFAs provide energy to B cells, memory T cells, and effector T cells by regulating metabolic pathways such as glycolysis, the TCA cycle, and β-oxidation to enhance ICI efficacy

Colonic anaerobes produce SCFA from undigested and absorbed carbohydrates or glycoproteins secreted by gut epithelial cells. Recently, the association between gut microbiota-derived SCFAs and nivolumab or pembrolizumab treatment in solid tumors has been confirmed [83]. SCFAs were found to be significant physical and chemical barriers that stimulate Paneth cells and goblet cells to produce AMPs and mucus to support the integrity of the intestinal mucosal barrier [84, 85]. SCFAs play a key role in complex gut microbial immune and metabolic networks, affecting the activity of immune cells and tumor cells (Fig. 4).

SCFAs inhibit the proliferation and induce the apoptosis of tumor cells. The mechanism of the synergy of SCFAs and ICI is a research hotspot. Butyric acid, the metabolite of *Faecalibaculum rodentium* PB1 (F. PB1) and *H. biformis*, acts as a histone deacetylase (HDAC) inhibitor [86]. It increased acetylation and inhibited calcineurin-mediated nuclear factor of activated T cells C3 (NFATc3) activation, which blocked tumor cell proliferation [86] (Fig. 4A). In addition, the SCFA propionic acid produced by *A. muciniphila* activated the cell cycle inhibitor p21 through G protein-coupled receptor 43 (GPR43) and downregulated inhibitor of apoptosis protein (IAP), which restrained cancer cell proliferation, induced apoptosis, and improved the antitumor effect of ICI [87] (Fig. 4A).

SCFAs improve antitumor immune responses. SCFAs can directly promote the antitumor cytotoxicity of CD8 T cells in vivo and in vitro [6, 88] (Fig. 4B). Butyrate produced by gut microbiota metabolism could directly boost the antitumor cytotoxicity of CD8^+^ T cells by inhibiting DNA binding 2 (ID2)-dependent IL-12 signaling [6]. Another study confirmed that valeric acid and butyric acid enhanced the function of mTOR as central cellular metabolic sensors and inhibited the activity of class I HDACs through metabolic and epigenetic reprogramming [88]. Moreover, this led to an increase in effector molecules such as CD25, IFNγ, and TNFα, which significantly promoted the antitumor effects of antigen-specific cytotoxic T lymphocytes (CTLs) and chimeric antigen receptor (CAR) T cells in melanoma and pancreatic cancer mouse models [88]. Both studies identified valeric acid and butyric acid as two SCFAs with therapeutic effects in cancer immunotherapy.

SCFAs provide energy for immune cells. SCFAs derived from the gut microbiota can regulate glycolysis, the tricarboxylic acid (TCA) cycle, the PPP, and fatty acid oxidation (FAO) of antitumor effector cells to increase the efficiency of ICI (Fig. 4C). First, butyrate, an SCFA, is converted to butyryl-CoA, undergoes β-oxidation (β-OX), and participates in the TCA cycle and oxidative phosphorylation (OXPHOS). These processes provide intestinal mucosal epithelial cell energy, improve intestinal villus structure, inhibit autophagy, and preserve intestinal homeostasis [89, 90]. Second, in memory T cells, butyric acid activated intracellular β-OX, promoting the TCA cycle and OXPHOS [91]. At the same time, acetyl-CoA produced by acetic acid promoted glycolysis by acetylating glyceraldehyde-phosphate dehydrogenase (GAPDH) [92]. Importantly, in effector T cells, SCFAs increase the number of mitochondria in cells and stimulate glycolysis and OXPHOS [93, 94]. Finally, SCFAs provide energy for B-cell differentiation into plasma cells, antibody production, and overall changes in cell metabolism [95].

### Anacardic acid

**Fig. 5:**
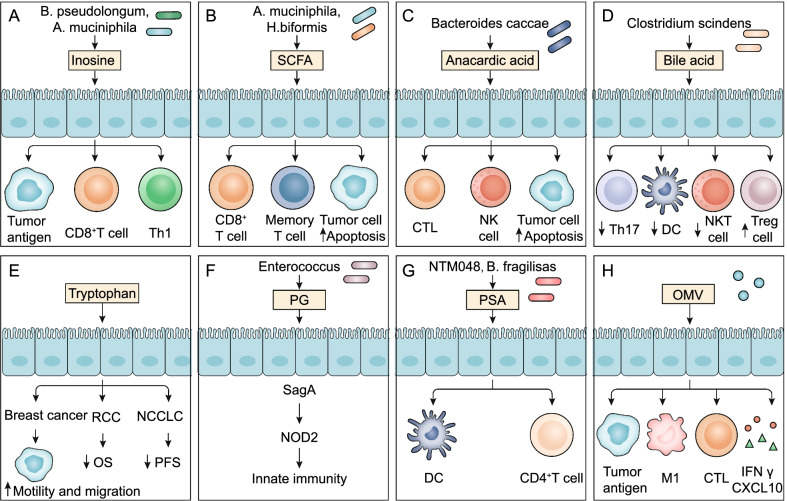
Microbiota-derived metabolites and other gut microbial signature modulation of antitumor immune responses to improve ICI efficacy. **A–E** Microbiota-derived metabolites target immune cells and tumor cells to modulate antitumor immunity. Inosine, SCFA, and anacardic acid promote antitumor immunity and ICI efficacy; bile acid and tryptophan attenuate antitumor immune responses. **F–H** Other gut microbial signature modulation of antitumor immune responses. PG, PSA, and OMV promote anti-tumor immune responses by regulating immune cells, tumor cells, and cytokines

In addition to inosine and SCFAs (Fig. 5A, B), anacardic acid also modulates antitumor immune responses (Fig. 5C). In the prospective study, metabolomics analysis of fecal bacteria from melanoma patients treated with ICI showed that *Bacteroides caccae* was significantly enriched, and levels of anacardic acid were greatly increased in the ICI response group (62-fold, P < 0.01) [96] (Fig. 5C). Anacardic acid triggers the classic activation pathway in macrophages by phosphorylating mitogen-activated protein kinases (MAPKs), thereby activating innate immunity [97]. Anacardic acid can also induce production of a neutrophil extracellular trap (NET), which promotes creation of tumor-infiltrating immune cells with macrophages, NK cells, and T lymphocytes to modulate adaptive immunity and antitumor immunity [98, 99]. Indeed, anacardic acid has been confirmed to have antitumor effects in some preclinical models [100]. For example, in breast cancer models, anacardic acid increased levels of tumor-infiltrated NK cells and CTLs and induced apoptosis of tumor cells [101] (Fig. 5C).

### Bile acid and tryptophan

Gut microbiota-derived metabolites, such as secondary bile acid or tryptophan, have shown immunosuppressive effects in some studies (Fig. 5D, E). Gut microbiota that produce bile acid metabolite 3-oxolithocholic acid as well as an abundant gut metabolite isolithocholic acid inhibit Th17 cell differentiation [102]. Furthermore, the secondary bile acid 3β-hydroxydeoxycholic acid exhibits weakened immunostimulatory properties when acting on DCs, thus inducing the expression of Foxp3, upregulating the number of Tregs, and promoting immune escape [103]. Feeding secondary bile acids or bile acid-metabolizing bacteria *Clostridium scindens* attenuated NKT cell-mediated liver-selective tumor inhibition [47]. In addition, IL-2 induced CD8^+^ T cell exhaustion and strong expression of tryptophan hydroxylase 1 by activating the STAT5-5-hydroxytryptophan (5-HTP)-AhR axis [104]. Tryptophan metabolites effectively facilitated the motility and migration of tumor cells in breast cancer [105, 106]. The increase in the serum kynurenine/tryptophan ratio was associated with worse overall survival in melanoma and RCC patients receiving nivolumab therapy [107]. Low plasma tryptophan metabolite-3-hydroxyphthalate levels were significantly associated with prolonged median progression-free survival (PFS) in patients with NSCLC [108].

## Other gut microbial signatures guide antitumor immune responses

### Peptidoglycan (PG) and polysaccharide (PSA)

Enterococcus expressed and secreted orthologs NlpC/p60 PG hydrolase SagA and could promote expression of the innate immune sensor protein nucleotide-binding oligomerization domain containing 2 (NOD2) and augment ICI antitumor efficacy [109] (Fig. 5F). Recognition of microbiota-derived PG in a nucleotide-binding oligomerization domain containing 1 (NOD1)-dependent manner could facilitate systemic innate immunity [110]. Furthermore, PSA produced by *Leuconostoc mesenteroides* strain NTM048 or *Bacteroides fragilis* acts as an immunostimulant to enhance the mucosal barrier and influence systemic immune responses [111, 112] (Fig. 5G). PSA can be recognized by DCs in the small intestine and activated CD4^+^ T cells to secrete cytokines, thereby promoting T cell proliferation, improving Th1/Th2 cell imbalance, and promoting lymphoid tissue formation [113] (Fig. 5G). TLRs, such as TLR9 and its agonist CpG-ODN, play an essential role in pathogen recognition and initiation of immune responses [114–118]. *Clostridium difficile* toxin A-bound DNA activated TLR9 signaling and the innate immune response [119].

### Outer membrane vesicle (OMV)

Microbiota-derived OMVs naturally secreted by bacteria can reprogram the TME and have been developed into tumor immunotherapeutic reagents, bacterial vaccines, adjuvants, and drug delivery carriers [120, 121]. OMVs express tumor antigens, inducing innate immune responses and antigen-specific T cell-mediated antitumor immunity; bioengineered bacterial OMVs expressing multiple tumor antigens can trigger a synergistic antitumor immune response [122] (Fig. 5H). OMVs with calcium phosphate (CaP) shells promoted cytotoxic T cell infiltration and M2 to M1 macrophage polarization, effectively improving the immunosuppressive TME [123] (Fig. 5H). Additionally, systematically administered bacterial OMVs specifically targeted and accumulated in the tumor bed and subsequently induced the production of the antitumor cytokines CXCL10 and IFN-γ to effectively augment antitumor immune responses [124] (Fig. 5H).

## Gut microbiota and ICI therapy toxicity

ICI therapy disrupts the host immune balance while killing tumor cells, which may result in immune-related colitis, immune-related pneumonia, and even life-threatening immune-related myocarditis. Several lines of evidence suggest that the role of the gut microbiota in immune-related adverse events (irAEs) is a double-edged sword. Certain gut bacteria may be protective against immunotherapy-induced toxicity. Mouse models have verified that *Bifidobacteria*, *Bacteroides fragilis*, and *Burkholderia cepacia* can ameliorate intestinal immunopathology in the context of anti-CTLA-4 therapy [17, 125, 126]. A prospective study demonstrated that increased representation of the *Bacteroidetes phylum* and microbial genetic pathways involved in polyamine transport and vitamin B biosynthesis was associated with developed resistance to ICI-induced colitis in metastatic melanoma patients treated with ipilimumab [127]. In contrast, some gut microbiota are associated with a high risk of ICI-induced toxicity. The enrichment of two microbes, *Lachnospiraceae spp.* and *Streptococcus spp.*, was associated with an increase in irAEs in melanoma patients treated with anti-PD-1 [128]. Interestingly, a distinct baseline of gut microbiota may also be associated with favorable anticancer response as well as ICI-induced toxicity. Among 26 patients with metastatic melanoma treated with ipilimumab, patients with *Faecalibacterium* and *Firmicutes* enrichment at baseline were prone to develop immunotherapy-induced colitis and enhanced ICI sensitivity simultaneously [49]. This is known as the efficacy–toxicity coupling effect in the context of ICI [126, 129, 130]. Gut microbes, such as *Bacteroides*, could also be a biomarker for predicting ICI therapeutic toxicity in advanced melanoma patients treated with combined CTLA-4 and PD-1 blockade [131]. The beneficial effect of FMT as a therapeutic modality for immunotherapy-induced toxicity in two patients has been demonstrated by reconstructing the gut microbiome and increasing the proportion of Tregs in the colonic mucosa [132]. These studies indicate that the gut microbiome has complex positive and negative effects on ICI-induced toxicity. More evidence is required before adequately filtering and manipulating the gut microbiota to augment the ICI response and attenuate irAEs.

## Therapeutic strategies utilizing the gut microbiome combined with ICI

**Fig. 6:**
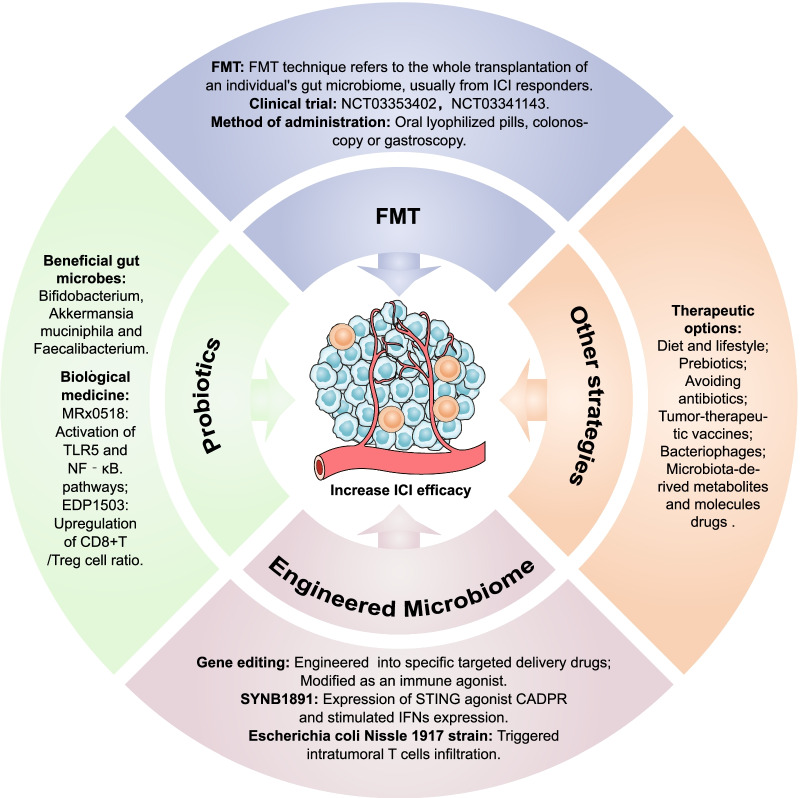
Therapeutic strategies utilizing the gut microbiome combined with ICI. Therapeutic strategies for manipulating gut microbiota include FMT, probiotics, engineered microbiome, and other strategies to increase ICI responses

Understanding the biological mechanisms of the gut microbiome and its metabolites on antitumor immunity and immunotherapy responses is essential for rationally manipulating microbial activities to improve ICI efficacy [133, 134]. The therapeutic strategies utilizing the gut microbiome combined with ICI are delineated in Fig. 6.

### Fecal microbiota transplantation

FMT treatment refers to the whole transplantation of an individual's gut microbiome, usually from ICI responders. FMT preparations can be administered directly by oral lyophilized pills or by colonoscopy or gastroscopy. FMT was initially used to treat *Clostridium difficile* infection resistant to traditional therapy [135]. Recently, several studies have indicated that FMT could augment the antitumor effect of ICI and overcome resistance to immunotherapy [31–33]. In a phase I clinical trial (NCT03353402), researchers performed FMT and reinduction anti-PD-1 therapy for ten melanoma patients who were unresponsive to PD-1 blockade [15]. The results showed that three of the ten patients showed tumor volume decline, including two partial responses (PR) and one complete response (CR) [15]. In the same period, another clinical trial focused on 15 melanoma patients resistant to anti-PD-1 therapy (NCT03341143), and the results showed that three patients showed PR after using FMT combined with pembrolizumab, and three patients had stable disease (SD) for more than 12 months [14]. The application of FMT is a novel approach for reversing ICI immunotherapy resistance and decreasing irAEs. Some clinical trials to evaluate the safety and efficacy of the combination of FMT with ICI treatment are underway (Table 2).

**Table 2:** Clinical trials of FMT modulate the efficacy and AEs of ICI www.clinicaltrials.gov

NCT number	Cancer types	*n*	Intervention	Outcome(s)	Stage	References
**Modulation of the gut microbiome to improve ICI efficacy**						
NCT04056026	Mesothelioma	1	FMT + Pembrolizumab	PFS	Phase 1	–
NCT04521075	Melanoma, NSCLC	50	FMT + Nivolumab	FMT-related AEs, ORR	Phase 1–2	–
NCT04130763	Gastrointestinal	10	FMT + Anti-PD-1	FMT-related AEs, ORR	Phase 1	–
NCT04116775	Prostate	32	FMT + Enzalutamide + Pembrolizumab	Anticancer effect	Phase 2	–
NCT04729322	dMMR CRC	15	FMT + Pembrolizumab–Nivolumab	ORR	Phase 1	–
NCT04577729	Melanoma	60	Allogenic FMT + ICI versus Autologous FMT + ICI	PFS	NA	–
NCT04758507	RCC	50	Donor FMT + ICI versus Placebo FMT + ICI	PFS	Phase 1–2	–
NCT04924374	NSCLC	20	Anti-PD-1 + FMT versus Anti-PD-1	Treatment safety and responses	NA	–
NCT04988841	Melanoma	60	MaaT013 + Ipilimumab + Nivolumab versus placebo + Ipilimumab + Nivolumab	AE, ORR	Phase 2	–
NCT03772899	Melanoma	20	FMT + Pembrolizumab/Nivolumab	Safety, ORR	Phase 1	–
NCT03341143	Melanoma	20	FMT + Pembrolizumab	ORR	Phase 2	[14]
NCT03353402	Melanoma	40	FMT + ICI	FMT-related AEs,	Phase 1	[15]
**Modulation of the gut microbiome to prevent ICI-related AEs**						
NCT04038619	RCC	40	Loperamide + FMT + ICI	FMT-related AEs, ICI-related diarrhea/colitis	Phase 1	–
NCT04163289	Renal cancer	20	FMT + Nivolumab/Ipilimumab	Immune-related colitis	Phase 1	–
NCT03819296	Solid tumors	800	FMT + Infliximab/Vedolizumab	FMT-related AEs, ICI-related colitis	Phase 1–2	–
NCT04883762	Solid tumors	10	FMT + ICI	FMT-related AEs, ICI-related diarrhea	Phase 1	–

AEs: adverse events; CRC: colorectal cancer; dMMR: mismatch-repair deficiency; FMT: fecal microbiota transplant; ICI: immune checkpoint inhibitor; n: number of patients; NA: not applicable; NSCLC: non-small cell lung cancer; ORR: objective responses rate; PFS: progression-free survival; and RCC: renal cell carcinoma

Despite the promising results of FMT therapy in patients treated with ICI, there are still concerns about its long-term safety. In 2019, it was reported that in two independent clinical trials, two patients developed bacteremia with extended-spectrum β-lactamase (ESBL)-producing *Escherichia coli* after receiving FMT from the same donor, and one patient died [136]. This study led the US Food and Drug Administration to issue a safety bulletin warning of the infection risk with FMT therapy. Furthermore, a recent retrospective cohort study of collected donor feces tested for multidrug-resistant organisms showed that 6 of 66 tested individuals (9%) were positive for multidrug-resistant organisms at any timepoint [137]. Therefore, periodic screening of donor feces should be performed to strictly limit the spread of organisms that may lead to adverse infection events, which is especially relevant for immunodeficient patients. Further clinical investigations enabling a better understanding of the source, transplantation procedure, and recipient phenotype of donor FMT are critical for successful ICI-FMT combination therapy.

### Probiotics

Probiotics are “live microorganisms which when administered in adequate amounts confer a health benefit on the host” [138]. Early clinical trials in patients with cancer mainly evaluated how probiotics modified microbiota composition or modulated antitumor immunity. A clinical trial in patients with breast cancer assessed the effects of the administration of probiotics (13 strains of beneficial bacteria) on CD8^+^ T cell infiltration in the TME (NCT03358511). Patients with CRC administered probiotics (*Bifidobacterium lactis* and *Lactobacillus acidophilus*) had an increased abundance of butyrate-producing bacteria in the tumor, mucosa, and feces (NCT03072641) [139]. Another trial demonstrated that patients treated with probiotics have lower IL-1b, IL-10, and IL-23A mRNA levels in the colonic mucosa (NCT01895530) [140]. Moreover, specific gut microbes, such as *Bifidobacterium* [16, 33, 51, 141], *Akkermansia* [31, 38], *Enterococcus* [15, 33, 141], *Faecalibacterium* [49, 142], and *Ruminococcaceae* [32], play the role of immune adjuvants in ICI immunotherapy. Several clinical trials to assess the safety and efficacy of probiotics combined with ICIs are underway (Table 3). For example, MRx0518 (*Enterococcus gallinarum* capsule) mainly relies on free flagellin to activate the TLR5 and NF‐κB signaling pathways and exert antitumor effects [143]. The phase I/II open-label clinical trial (NCT03637803) explored the synergistic effects of the oral probiotic MRx0518 in combination with pembrolizumab in NSCLC, RCC, bladder cancer, or melanoma, and the results have not yet been published. EDP1503 is a novel adjuvant for the immunotherapy of cancer-based *Bifidobacterium*. Clinical trials (NCT03775850) showed that EDP1503 in combination with pembrolizumab was safe and well tolerated, and biomarker studies found that EDP1503 works by upregulating the ratio of CD8^+^ T cells/Treg cells [144].

**Table 3:** Clinical trials of probiotics and engineered microbiomes in combination with ICIs in cancer treatment www.clinicaltrials.gov

NCT number	Cancer types	*n*	Microbiome-based therapy	Intervention	Stage	References
NCT03775850	Solid tumors	120	EDP1503 (*Bifidobacterium* strain)	EDP1503 (capsules) + Pembrolizumab	Phase 1–2	[144]
NCT03637803	Solid tumors	132	MRx0518 (*E. gallinarum* strain)	MRx0518 (capsules) + Pembrolizumab	Phase 1–2	–
NCT05107427	Urothelial carcinoma	30	MRx0518 (*E. gallinarum* strain)	MRx0518 (capsules) + Avelumab	Phase 2	–
NCT03686202	Solid tumors	65	MET-4 (Mixture of pure live cultures of intestinal bacteria)	MET (capsules) + ICI	Phase 1	–
NCT04601402	Solid tumors	93	GEN-001 (*Lactococcus lactis* strain)	GEN-001 (capsules) + Avelumab	Phase 1	–
NCT04187404	Adrenal tumors	60	EO2401 (Vaccines of microbial-derived peptide homologous to tumor-associated antigens)	EO2401 + Nivolumab	Phase 1–2	–
NCT04116658	Glioblastoma	52	EO2401 (Vaccines of microbial-derived peptide homologous to tumor-associated antigens)	EO2401 + Nivolumab	Phase 1–2	–
NCT03829111	Renal cell carcinoma	30	CBM588 (*Clostridium butyricum* probiotic)	Nivolumab + Ipilimumab versus CBM588 (capsules) + Nivolumab + Ipilimumab	Phase 1	[145]
NCT04167137	Solid neoplasm, lymphoma	70	SYNB1891 (Engineered *E. coli*)	SYNB1891 (intratumoral injection) + Atezolizumab	Phase 1	–
NCT04208958	Solid tumors	54	VE800 (11 commensal bacterial strains)	VE800 + Nivolumab	Phase 1–2	–
NCT03817125	Melanoma	14	SER-401 (Defined bacterial consortia)	SER-401 (capsules) + Nivolumab versus Placebo + Nivolumab	Phase 1	–

ICI: immune checkpoint inhibitor; n: number of patients

### Engineered microbiomes

With the development of bacterial genetic engineering technology, it is possible to improve antitumor responses to ICI by modifying gut microbiota or metabolites. Genetically engineered drugs have specificity that FMT cannot achieve. To date, genetically attenuated, nutrient deficient, and inducible *Escherichia coli* [146], *Bifidobacteria* [147], *Listeria* [148], and *Salmonella* [148] have been transformed and have shown antitumor effects in preclinical models of intravenous, intratumoral, and oral administration routes [149]. Guo et al. [150] made use of the abilities of several strains, including *Salmonella* and *Clostridium*, to elicit specific targeting in solid tumors, making them ideal carriers for the delivery and induction of immune stimulants to delay tumor growth and metastasis. In addition, SYNB1891 is a dual innate immune agonist designed based on the biology of *E. coli*. SYNB1891 has been modified to express the STING agonist cyclic adenosine diphosphate ribose (CADPR), which can stimulate expression of IFNs and achieve antitumor effects [151]. Clinical trials exploring the safety and efficacy of SYNB1891 in combination with atezolizumab in advanced solid tumors are ongoing (Table 3). Furthermore, L-arginine enhances T cell survival and antitumor activity by modulating T cell metabolism [152, 153]. The engineered probiotic *Escherichia coli* Nissle 1917 strain colonizes tumor sites and continuously converts the metabolite ammonia to L-arginine in the tumor bed [154]. Intertumoral injection of this strain in mice increased the intracellular L-arginine concentration, triggered intratumoral CD4^+^ T cell and CD8^+^ T cell infiltration, and exerted synergistic antitumor effects when combined with anti-PD-L1 [154]. These results show that engineered microbial therapies enable metabolic modulation of the TME to enhance the efficacy of immunotherapies.

### Other strategies

Therapeutic strategies involving the manipulation of gut microbiota to enhance ICI efficacy also include adjusting diet and lifestyle, taking prebiotics, and avoiding antibiotics [155–158]. Additionally, the discovery of tumor cross-antigens and gut microbiota-derived immune activators provides insights into the development of tumor-therapeutic vaccines such as Ty21a [159, 160], JNJ-64041809 [161], and VXM01 [162]. EO2401 is a microbial-derived polypeptide drug with a structure homologous to that of tumor-associated antigens. The clinical trial of EO2401 combined with nivolumab is ongoing in glioblastoma multiforme (GBM) and adrenocortical carcinoma patients (Table 3). It is noteworthy that oral administration of microbiome-derived compounds, such as bacteriophages and microbial metabolites, may be more practical and precise than administration of full or unique live bacteria transplants [70, 163]. Indeed, bacteriophages as therapeutic strategies could modulate the gut microbiota, immunity, and TME [70, 164–166]. Importantly, the SCFA valproic acid (VPA, 2-propylpetanoic acid) is a type of microbiota-derived metabolite. Clinical trials to assess the safety and efficacy of VPA in combination with ICI in patients with solid tumors are already underway (NCT02446431, NCT01106872, NCT02624128).

## Further directions

We have gained insights into the potential mechanisms of gut microbiome influences on the antitumor immune response and ICI efficacy and into their use in therapeutic strategies from preclinical and clinical research. In the future, we still need to consider the following factors. First, the accuracy of the findings of the microbiome influence on ICI efficacy should be demonstrated. The effects of gut microbiota on tumor ICI therapy are multifaceted and even bidirectional. We should accurately classify favorable and unfavorable microbiome features that target immune cells or pathways and fully understand their effects in different TMEs. Second, this approach facilitates the improved precision of therapeutic strategies. Understanding the microbiota-metabolite-immune axis helps us directly manipulate specific immune-stimulating metabolites or compounds derived from gut microbiota, rather than whole or unique live bacteria transplants, to improve the ICI response. In addition, the effects of other microbiota-derived molecules that promote the antitumor immune response may become a future direction of preclinical or clinical research on ICIs. Third, gut microbiota may act as a predictive biomarker for ICI efficacy or safety. A patient's microbiota data could be combined with those of other known related predictive markers, such as PD-L1 expression and tumor mutation load, to predict immunotherapy's potential efficacy or adverse events. Finally, the effectiveness of clinical trials should be improved. In human clinical trials, it is necessary to comprehensively monitor the factors influencing the gut microbiome, such as diet, probiotics, antibiotics, drugs, mental health, host immune system, host genetics, geographical factors, tumor type, and stage. Notably, multilevel and multidimensional research designs integrating microbiology, genetics, immunology, metabolomics, molecular pathology, and epidemiology will become a part of personalized cancer therapy in the future.

## Conclusion

In conclusion, the gut microbiome cross talk with innate and adaptive immune cells occurs, augmenting the intermediate effects of innate immune cells, enhancing the antitumor effect of adaptive immune cells, and increasing the immunogenicity of tumor cells, which reprograms the immunity of the TME and ameliorate ICI responses. Notably, microbiota-derived circulating metabolites modulate multiple human physiologies and spread from their original location in the gut to mediate local and systemic antitumor immune responses to promote ICI efficiency. Therapeutic strategies utilizing gut microbiota combined with ICI, such as appropriate antibiotic selection, probiotic intake, FMT, and bacterial genetic engineering, may provide novel possibilities for gut microbiota and their metabolites to become excellent adjuvants for ICI. Further understanding the mechanisms of synergy between ICI and the gut microbiome and accurate identification of immunostimulant and immunosuppressive strains or pathways is expected to enable the development of more effective combination therapy strategies for ICI and the advancement of precision medicine strategies.

## Data Availability

Not applicable.

## Abbreviations

5-HTP: 5-Hydroxytryptophan
ACT: T cell transfer
*A.** muciniphila*: *Akkermansia muciniphila*
A_2A_R: Adenosine 2A receptor
*B.** pseudolongum*: *Bifidobacterium pseudolongum*
CADPR: Cyclic adenosine diphosphate ribose
CaP: Calcium phosphate
CAR: Chimeric antigen receptor
CpG-ODN: CpG-oligodeoxynucleotide
CR: Complete response
CRC: Colorectal cancer
CTLA-4: Cytotoxic T lymphocyte-associated antigen-4
CTLs: Cytotoxic T lymphocytes
DCs: Dendritic cells
dMMR: Mismatch-repair deficiency
DTR: Diphtheria toxin receptor
ESBL: Extended-spectrum β-lactamase
F. PB1: *Faecalibaculum rodentium* PB1
FA: Fatty acid
FAO: Fatty acid oxidation
FMT: Fecal microbiota transplantation
GAPDH: Glyceraldehyde-phosphate dehydrogenase
GBM: Glioblastoma multiforme
GPR43: G protein-coupled receptor 43
HCC: Hepatocellular carcinoma
HDAC: Histone deacetylase
HLA: Human leucocyte antigen
IAP: Inhibitor of apoptosis protein
ICI: Immune checkpoint inhibitors
ID2: Inhibiting DNA binding 2
IFN: Interferon
IL: Interleukin
IrAEs: Immune-related adverse events
LAG3: Lymphocyte-activating gene-3
Macs: Macrophages
MAIT: Mucosal-associated invariant T
MAPKs: Mitogen-activated protein kinases
MHC: Major histocompatibility complex
NA: Not applicable
NCR: Natural cytotoxic receptor
NET: Neutrophil extracellular trap
NFATc3: Nuclear factor of activated T cells C3
NK: Natural killer
NOD1: Nucleotide-binding oligomerization domain containing 1
NOD2: Nucleotide-binding oligomerization domain containing 2
NSCLC: Non-small cell lung cancer
OMV: Outer membrane vesicle
ORR: Objective response rates
OXPHOS: Oxidative phosphorylation
PBMCs: Peripheral blood mononuclear cells
pCREB: Phosphorylation of cAMP responses element-binding protein
PD-1: Programmed cell death 1
PD-L1: Programmed cell death ligand-1
PFS: Progression-free survival
PG: Peptidoglycan
PHA: Phytohemagglutinin
PNP: Purine nucleoside phosphorylase
PPP: Pentose phosphate pathway
PR: Partial responses
PSA: Polysaccharide
PSMB4: Proteasome subunit beta type-4
RCC: Renal cell carcinoma
SCFAs: Short-chain fatty acids
SD: Stable disease
SIY: SIYRYYGL
STING: Stimulator of interferon genes
SVY: SVYRYYGL
TCA: Tricarboxylic acid
TCR: T cell receptor
Th1: T helper 1
TLR: Toll-like receptor
TME: Tumor microenvironment
TMP1: Tape measure protein 1
TNF: Tumor necrosis factor
β-OX: β-Oxidation
